# Intermediate care: for better or worse? Process evaluation of an intermediate care model between a university hospital and a residential home

**DOI:** 10.1186/1472-6963-5-38

**Published:** 2005-05-24

**Authors:** Thomas Plochg, Diana MJ Delnoij, Tineke F van der Kruk, Tonnie ACM Janmaat, Niek S Klazinga

**Affiliations:** 1 Department of Social Medicine, Academic Medical Centre / University of Amsterdam, Meibergdreef 9, Amsterdam, The Netherlands; 2 Nivel Netherlands Institute for Health Services Research, Drieharingstraat 6, Utrecht, The Netherlands; 3 Department of Geriatrics, Academic Medical Centre / University of Amsterdam, Meibergdreef 9, Amsterdam, The Netherlands; 4 Medical Board, Academic Medical Centre / University of Amsterdam, Meibergdreef 9, Amsterdam The Netherlands

## Abstract

**Background:**

Intermediate care was developed in order to bridge acute, primary and social care, primarily for elderly persons with complex care needs. Such bridging initiatives are intended to reduce hospital stays and improve continuity of care. Although many models assume positive effects, it is often ambiguous what the benefits are and whether they can be transferred to other settings. This is due to the heterogeneity of intermediate care models and the variety of collaborating partners that set up such models. Quantitative evaluation captures only a limited series of generic structure, process and outcome parameters. More detailed information is needed to assess the dynamics of intermediate care delivery, and to find ways to improve the quality of care. Against this background, the functioning of a low intensity early discharge model of intermediate care set up in a residential home for patients released from an Amsterdam university hospital has been evaluated. The aim of this study was to produce knowledge for management to improve quality of care, and to provide more generalisable insights into the accumulated impact of such a model.

**Methods:**

A process evaluation was carried out using quantitative and qualitative methods. Registration forms and patient questionnaires were used to quantify the patient population in the model. Statistical analysis encompassed T-tests and chi-squared test to assess significance. Semi-structured interviews were conducted with 21 staff members representing all disciplines working with the model. Interviews were transcribed and analysed using both 'open' and 'framework' approaches.

**Results:**

Despite high expectations, there were significant problems. A heterogeneous patient population, a relatively unqualified staff and cultural differences between both collaborating partners impeded implementation and had an impact on the functioning of the model.

**Conclusion:**

We concluded that setting up a low intensity early discharge model of intermediate care between a university hospital and a residential home is less straightforward than was originally perceived by management, and that quality of care needs careful monitoring to ensure the change is for the better.

## Background

Due to technological developments, better communication facilities and further differentiation and specialisation of professionals, patient care is increasingly provided outside hospitals. Financiers, governments and patients advocate this development, as they consider outpatient care to be more efficient and patient centred [1].

This shift has repercussions for chronically ill and elderly patients with complex care needs. First, it is questionable whether optimal care for these patients can be delivered in an outpatient setting. They need sufficient time to recover and are often in frail health [2]. Early hospital discharge can present dangers for them. Moreover, alternatives are often scarce, as the capacity of long-term care facilities is either limited or too expensive. What is known as 'bed-blocking' occurs when patients whose medical treatment has been completed cannot be discharged because of poor continuation of care outside the hospital [3].

Second, these patients often need care from health professionals in multiple settings. Consequently, patient care journeys encompass multiple transitions from one setting to another [4]. Systems of care often fail to organise these transitions, thus influencing the quality of care [5-7].

Bearing these issues in mind, a plethora of care models have been developed to substitute hospital inpatient care and to improve transitional care [8]. One model can be described by the term 'intermediate care'. This model refers to a range of services intended to bridge acute, primary and social care. It is considered to serve goals like reducing the length of hospital stays, preventing hospital admissions and readmissions, improving transitions from hospital to consecutive settings, and retaining people's independence as long as possible [9].

Still, the benefits and transportability of intermediate care are ambiguous [9-12]. This can be attributed to the blurred definition of the concept and the wide range of services labelled as such. Models differ in focus, setting, case mix, staffing, professionals involved, commissioning and context. Hence, it is quite difficult to define the concept, to identify best practices and to compare different settings. Against this background, it is argued that the 'black-box' of intermediate care (i.e. the processes of care) should be opened [13]. Process evaluations are therefore warranted and promoted [14,15].

This paper provides such a process evaluation of an intermediate care model. The aim was to provide management with information to assess and improve the quality of care. Even so, it should also provide more generalisable insights into the accumulated impact of the model.

In the Dutch health care system, a distinction is made between the financing and organisation of acute and long-term care. Acute care is financed either by social health insurance (covering about 60% of the population) or private health insurance (covering about 40% of the population). Compulsory national health insurance covers the whole population for long-term care. Health care providers organise their services in networks that are covered by either scheme. Cooperation and coordination of care takes place predominately within the acute care sector.

But health care delivery is fragmented in the transition from acute to long-term care. To bridge the gap, various intermediate care models are being set up in hospitals, nursing homes and residential homes [16,17]. However, due in part to the division of the insurance schemes, financing is often ad hoc and available only for the duration of a single project.

The model evaluated in this study eventually became structurally embedded in the local health system of the South-eastern Amsterdam district. The Henriëtte Roland Holst House (HRHH), a residential home, and the Academic Medical Center of the University of Amsterdam (AMC) agreed to establish a low intensity early discharge model of intermediate care encompassing transitional care as well as a transfer unit. This model focuses exclusively on all AMC patients who no longer require hospital treatment but are not healthy enough to be discharged to their home situations. It supplements two more intensive rehabilitation models of intermediate care in nursing homes for stroke and orthopaedic AMC patients. Table 1 describes the model and its local context.

**Table 1:** Characteristics of the intermediate care model

Focus	Starting a 'transfer unit' in a residential home for AMC patients whose medical treatment has been completed, but are unfit to be discharged to their homes. The unit should serve as a substitute hospital ward that relieves the problem of 'bed-blocking' in the AMC and improves transitional care to the home situation.
Admission criteria	All AMC patients are eligible for admission to the transfer unit if they meet the following criteria:
	- Patient is medically stable and curative treatment has been completed;
	- Patient needs care that can be delivered by one nursing assistant;
	- Patient is not eligible for other regular care services and cannot go home;
	- Patient is insured;
	- Patient does not need daily care and/or intensive physical therapy;
	- Patient is not a drug addict, terminally ill or comatose, and does not have AIDS;
	- Patient does not exhibit disturbing behaviour if he or she is a psychiatric or psychogeriatric patient.
	- Patient has an official indication for discharge to a consecutive setting.

Transitional care	Three AMC liaison nurses control, plan and coordinate all transitions of AMC inpatients to the transfer unit systematised by agreed discharge procedures. The nursing home physician, occupational therapist, the liaison nurse and the head (an RN) assess whether an AMC patient will be admitted to the transfer ward.

Setting	20 transfer beds located in 10 rooms. The unit was established outside the AMC in a residential home in the South-eastern Amsterdam district. This institution accounts for 110 residential home places, 7 places for day care, 4 community health beds and 218 apartments for assisted living.

Staffing	Head of the transfer unit 1.0 FTE; nursing home physician 0.33 FTE, registered nurses 0.89 FTE; liaison nurses 0.5 FTE; occupational therapist 0.5 FTE; licensed practice nurses 11.61 FTE. Two physiotherapists with a practice in the residential care home are directly available for patients of the transfer unit. An AMC geriatric nursing specialist attends multidisciplinary meetings once a week.

Context	The AMC and Henriëtte Roland Holst House are located in the South-eastern Amsterdam district. This region accounts for approximately 85,000 residents of whom 7,000 (8%) are older than 65, and 61% belong to an ethnic minority. A number of institutions in the region provide care for the elderly: 1 AMC, 1 nursing home, 4 residential homes, 1 public home-care agency, 1 public health agency, 1 social care agency, 5 primary care centres and 1 institution for psychiatric care.

Commissioning	The local public insurer structurally finances the transfer unit. The annual budget is 758,205 euros. Transitional care is financed by the AMC budgets.

## Methods

### Aim

The intermediate care model was evaluated at the request of the collaborating institutions. During the course of 2000, both partners expressed concerns about how the model was functioning, and needed information to make informed decisions on how to improve the quality of care. This implied that the evaluation should be conducted from a 'managerial evaluation perspective' [18]. The following questions were addressed: 1) Is the patient population admitted to the model in accordance with the *ex ante *expectations of key players and staff members? 2) How does the model ensure quality of care?

### Design

A process evaluation based on quantitative and qualitative methods was considered the most appropriate and feasible way of answering both questions. Registration forms and patient questionnaires were used to quantify the patient population. Semi-structured interviews were conducted to explore the expectations and experiences of the key players and staff members, as well as to describe how the quality of care is ensured. The project proposal was reviewed by the medical ethical committee of the AMC in August 2000 and was considered not in need of formal approval according to the Dutch legislation on experiments with human beings. There were no ethical objections raised against the study.

### Patient questionnaires and registration forms (quantitative analyses)

#### Sample

Initially, all candidates (n = 189) for admission to the transfer unit between 1 October 2000 and 31 October 2001 were in the study. RNs working in the AMC wards selected candidates after consulting the individual patients and their families. Liaison nurses at the AMC collected the submitted applications and sent them to the transfer unit for assessment. Using formalised admission criteria (Table 1), the nursing home physician assessed whether a candidate could be admitted to the transfer unit. 162 out of 189 (85.7%) candidates met the admission criteria and 27 out of 189 (14.3%) did not. As researchers, we did not interfere in this process.

**Figure 1:**
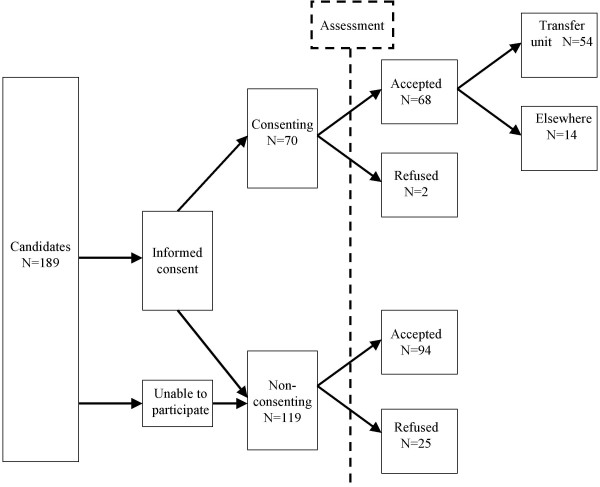
Flow chart

During the assessments, we requested informed consent from patients participating in the study. There were two significant differences between consenting (n = 70) and non-consenting patients (n = 119). The age distribution differed significantly between both groups (chi^2 ^= 12.51; p = .03), and the youngest and oldest patients were less willing to participate. Moreover, consenting patients were less likely to be refused for admission than those who did not consent. Just 2 out of 70 consenting patients (3%) were refused, while this rate was 27 out of 119 (21%) for non-consenting patients. This selective non-response can be explained by health status. Patients who did not meet the admission criteria were more seriously ill and less inclined to participate in the study. Of the 68 positively assessed patients who consented, 54 were actually admitted. During the assessment period 2 patients died and 12 were discharged to another destination. The sample is shown in figure 1.

#### Data collection

Two registration forms and two patient questionnaires measured process parameters. The first form, filled in before the assessment procedure was started, recorded 'medical diagnosis', 'reason for hospital admission', 'reason for application' (rehabilitation, waiting for admission to a nursing home, waiting for admission to a residential home, oncology, wound therapy, other), and 'name of the submitting AMC ward'. After the assessment procedure 'refusal or admittance to the transfer unit', 'date of hospital admission', 'date of hospital discharge', 'discharge destination' (transfer unit, home, nursing home, residential home, other) were registered.

The second form was filled out at discharge from the transfer unit. In this form 'destination of discharge' (home with or without home care, nursing home, residential home, hospital, other), 'experienced burden of care in relation to the expected burden of care based on the application' (the head of the unit gave his or her assessment), 'whether the patient was in the appropriate place' (the head of the unit gave his or her assessment), and 'care delivery problems experienced' were registered.

During the assessment procedure, the patients filled out two questionnaires, assisted by a researcher if necessary: the 36-item short-form health survey (SF-36) and the Groningen Activity Restriction Scale (GARS). The SF-36 includes one multi-item scale measuring eight health concepts: 1) limitations in physical activities because of health problems; 2) limitations in social activities because of physical or emotional problems; 3) limitations in usual role activities because of physical health problems; 4) bodily pain; 5) general mental health (psychological distress and well-being); 6) limitations in usual role activities because of emotional problems; 7) vitality (energy and fatigue) and 8) general health perceptions [19,20]. The GARS aims at measuring both the ADL and instrumental ADL disability in community-based studies with respect to the aid and services provided by professional home help and district nursing agencies [21,22].

#### Data analysis

We analysed the quantitative data using SPSS 10.1. We used T-tests and chi-squared test statistics to assess significance.

### Semi-structured interviews (qualitative analyses)

#### Sample

We interviewed 21 key players and staff members selected 'purposively' for their positions, disciplines and institutions. All disciplines involved were represented in the study. In addition, nursing assistants were selected on the basis of gender, age, ethnicity and work experience. It was not feasible to interview them all. This approach resulted in the sample presented in table 2.

**Table 2:** Interviewed professionals

Respondent	Position	Institution
Nr 1.	General manager	HRHH
Nr 2.	Director of integrated care	AMC
Nr 3.	Head of care department	HRHH
Nr 4.	Chair board of directors	HRHH
Nr 5.	Chair medical specialist staff	AMC
Nr 6.	Nursing home physician	HRHH
Nr 7.	Liaison nurses / head discharge unit	AMC
Nr 8.	Liaison nurse	AMC
Nr 9.	Liaison nurse	AMC
Nr 10.	Geriatric nurse specialist	AMC
Nr 11.	Liaison nurse / occupational therapist	HRHH
Nr 12.	Registered nurse internal medicine	AMC
Nr 13.	Registered nurse / Head of the transfer unit	HRHH
Nr 14.	Occupational therapist	AMC
Nr 15.	Physical therapist	HRHH
Nr 16.	Physical therapist	HRHH
Nr 17.	Nursing assistant transfer unit	HRHH
Nr 18.	Nursing assistant transfer unit	HRHH
Nr 19.	Nursing assistant transfer unit	HRHH
Nr 20.	Nursing assistant transfer unit	HRHH
Nr 21.	Nursing assistant transfer unit	HRHH

#### Data collection

**Figure 2:**
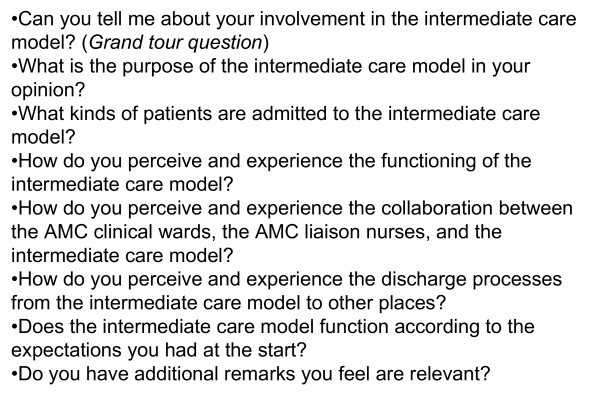
Interview guide

We interviewed the respondents at their places of work using an interview guide (see figure 2), which was developed around the research questions. During the interviews, the guide was used in an informal and flexible way in order to prevent the researchers from imposing their own preconceptions. The interviews took approximately one hour each; they were recorded and later transcribed. One researcher coded the transcripts and wrote memos to systematise the analysis. We completed the data collection after finishing 21 interviews – all disciplines working with the model were then represented and no new findings were expected.

#### Data analysis

We used open and framework approaches to analyse the interviews. Building on respondents' perceptions, we conceptualised the dynamics underlying the admittance of patients to the transfer unit. To interpret quality assurance activities and their implementation, a theoretical framework was explicated. On the basis of a typology of quality systems consisting of five elements (structural assets, allocation of responsibilities, protocols, information transfer and monitoring/feedback cycles) we identified existing quality assurance activities in the model. We contrasted respondents' notions on the implementation of the model with recent knowledge on effective implementation [23,24].

### Rigour

To monitor and consider the rigour of the interviewing process, we used several strategies to rule out validity threats [25]. First, due to our sampling strategy we were able to identify respondents who gave socially desirable answers. We noticed that three of them were much too positive about the functioning of the transfer unit and were defensive in their responses. Second, we conducted member checks by asking respondents to validate transcripts and interpretations of their interviews. Respondents had few actual corrections, which we adopted without discussion. Third, we aimed for 'triangulation'. We verified respondents' essential statements by contrasting them with our quantitative data, local documents and/or international and Dutch literature. Finally, we solicited feedback from a variety of senior and other researchers (*peer review*). The researchers in the team systematically monitored the data collection, analysis and emerging findings. Colleagues at the Department of Social Medicine and the Dutch National Institute of Health Services Research reviewed earlier drafts of the manuscript.

## Results

### Foreseen versus actual/perceived patient population

The target population was described in the initial intermediate care model [26]. AMC patients whose medical treatment had been completed but who were unfit to be discharged to their homes – the bed-blockers- were eligible for admission to the unit. Admission criteria further specify this target population (table 1). Quantitative as well as qualitative data were used to verify whether members of this target population were actually admitted to the unit.

### The quantitative profile of the patient population

During the 13 months of the study, 189 candidates were assessed (table 3). The majority were female, single and older than 65. Apart from this, the profile of these candidates was more heterogeneous. Although the most prevalent diseases were cardiovascular diseases and cancer, candidates suffered from a variety of different diseases. This was also shown by the diversity of the submitting clinical wards and the various reasons for application. A relatively small number of candidates were waiting for placement in a nursing home (n = 33) or residential home (n = 3) and could be identified as bed-blockers.

**Table 3:** Characteristics of assessed candidates

Process parameter	Outcome
Gender	Male n = 72 (38.3%)
	Female n = 117 (61.7%)

Age distribution	< 65 years n = 34 (18.3%)
	65–69 years n = 21 (11.3%)
	70–74 years n = 19 (10.2%)
	75–79 years n = 40 (21.5%)
	80–84 years n = 33 (17.7%)
	> 85 years n = 39 (21.0%)
	missing n = 3

Home situation	Living alone n = 160 (84.7%)
	Living with a partner n = 29 (15.3%)

Medical diagnoses	Cardiovascular diseases n = 44 (23.3%)
	Cancer n = 37 (19.4%)
	Other n = 108 (57.3%)

Reasons for application	Recovery after surgery n = 30 (17.4%)
	Rehabilitation n = 65 (37.8%)
	Waiting for a nursing home n = 33 (19.2%)
	Waiting for a residential care home n = 3 (1.8%)
	Oncology therapy n = 10 (5.8%)
	Other n = 31 (18.0%)
	Missing n = 17

The 'Health-related quality of life' (SF-36) and the 'Activities of daily living' (GARS DL-scale) were filled out by 59 of the 70 participating patients. Their scores were comparable with those of a population in a British geriatric day hospital and a population in a Dutch community GP ward for recuperating elderly people [27,28].

The 70 patients participating in the study had an average length of stay (LoS) in the AMC of 31.8 days (range 3–109; stdv 22.2). The 54 out of the 70 patients participating in the study who were actually admitted to the transfer unit stayed there for an average of 46.5 days (range 0–340). This average lies within the maximum of three months LoS. Looking back on their stays, the head of the transfer unit considered 12 of the 54 patients (22.2%) to have needed more care than had been expected, and 5 patients (9.3%) to have needed less care than had been expected. For 6 of the 12 more serious patients (11.1%) and for 3 of the 5 less serious ones (5.6%), the head also considered the transfer unit to have been inappropriate. The final discharge destination was home (n = 18; 33.3%), home with home care (n = 18; 33.3%), hospital (n = 6; 11.1%) or elsewhere (n = 12; 22.2%).

### A more complex patient load than anticipated

Respondents felt that a patient population with a heavier burden of care than anticipated had been admitted to the transfer unit. Respondents experienced a limited inflow of bed-blockers:

The focus of the model was to reduce the problem of bed-blocking in the AMC. Now we notice we admit hardly any bed-blockers to the transfer unit (…). In practice it seems there are no bed-blockers in the AMC. (respondent 10)

This perception was difficult to verify in the quantitative data. Based on the discharge destinations, just 36 out of 189 candidates (19%) could be considered as bed-blockers. This seems to be opposed by the average LoS in the AMC of 31.8 days, which is three times the average LoS of patients in the AMC in 2001 (9.4) [29]. However, the high standard deviation (22.2) and the median of 24 indicate that outliers are increasing the average LoS. Moreover, average LoS is an indicator of bed-blocking, not a valid measure.

To support their perception, respondents put forward two explanations. First, respondents felt the target population envisioned in the design of the intermediate care model either did not exist in the AMC or was smaller than expected. An initial assessment of the size of the target population did not take place. This implies that the relevance of the intermediate care model may have been overestimated from the outset:

You instinctively know the size of this patient group (…). As far as I know, there were no data available [during the planning of the transfer unit]. (respondent 8)

Second, respondents suggested that the assessment procedure took too long. The model is only appropriate during a short period of patients' care episodes because their care needs vary. Therefore, the transition from the AMC to the unit must be flexible and fast, and this was not achieved in practice. The period between application and discharge took on average 8.7 days (range 1–39). Moreover, in the study group, 14 out of 68 patients meeting the admission criteria were not discharged to the transfer unit. In the meantime, patients died (n = 2), went home (n = 5) or were admitted to another institution (n = 7). This implies that 20% of the inflow was cancelled during transitional care processes.

Respondents considered the main reason for delays to be the limited availability of the nursing home physician (only three afternoons a week), whose authorisation was needed for admission. Another reason mentioned for delays was discharge planning in the AMC. Time was sometimes lost waiting for the medical application/referral forms.

Due to the limited inflow of targeted patients, filling transfer beds became problematic. To avoid empty beds, respondents felt that admission criteria had been applied subjectively. In their opinion, restrictive application of inclusion criteria was not in the financial interests of the collaborating institutions:

There is a negative spiral nobody in health care can ignore. (...) On the one hand you must deliver a certain volume of care. On the other hand you have your human resources. When you fail delivering this volume of care you will loose personnel, as you earn not enough money. (...) What happens? You can admit even less patients. So, you must compromise. (respondent 6)

The HRHH maximised production to prevent budget reductions. The AMC optimised the turnover of patients to be in a better position for the annual budget negotiations with insurers. These inverse incentives may have resulted in admitting patients with a heavier burden of care to the transfer unit:

I think the patient population admitted to the transfer unit has a heavier burden of care than was originally expected. (respondent 8)

The medical aspects become more serious. Although the patients' medical treatment has been completed, their health is still frail. (respondent 6)

This perceived pattern was partly supported by quantitative data. According to the official hospital statistics bed-blocking days increased from 2,818 days in 2000 towards 3,315 days in 2001 while bed-occupancy rates decreased from 61.5% in 2000 to 57.5% in 2001 [29]. This may indicate that hospital discharge of bed-blockers is postponed to maximise bed-occupancy rates, which supports the hypothesis of the respondents. However, the severity of the self-reported health status of the patients (SF-36) did not change during the study.

### Quality of care

Almost all respondents felt there was insufficient quality of care assurance and questioned the functioning of the model:

It works, but if a few things go wrong it doesn't work anymore – then the quality of care goes down fast. (respondent 11)

It doesn't function like it should, but in the past it was worse. (respondent 17)

Although we cannot verify these quotes, we assume that the intermediate care model functions poorly because it was mentioned by the majority of the respondents. Two key players (respondents 6, 10) gave an overall explanation for the poor functioning of the model. They highlighted contextual differences between the HRHH and the AMC as the main stumbling blocks. Setting up and implementing intermediate care requires a certain level of know-how and expertise. In the AMC, these kinds of requirements could easily be met, while in the HRHH they could not. Initiators took this difference for granted, thereby overestimating the organisational capacity of the HRHH:

I don't think all of the consequences [of setting up a transfer unit] were foreseen...There was no experience available of caring for these patients in another setting. (respondent 6)

This overall explanation is supported by respondents' thoughts on the implementation and on the nursing staff mix. Because the interviews were planned to take place throughout the study period, we noticed during the course of 2001 that all planned working processes and quality assurance activities were put into place. The respondents interviewed early in the study reported fewer activities than those interviewed later. Table 4 presents the processes and activities categorised in the five dimensions of a quality system. Although these activities confirmed the existence of quality of care assurance practices, the rather late implementation suggested otherwise. Respondents said that people don't work enough according to the agreed working processes and quality assurance practices, and concluded the implementation process was flawed:

**Table 4:** Quality assurance activities in the transfer unit

Structural assets	- Description of required staff
	- Facilities
Allocation of responsibilities	- Job descriptions
	- Job assessment interviews

Protocols	- Description of the target population
	- Admission criteria
	- Discharge criteria
	- Routing of the patients using a flow chart
	- Nursing care plans

Information transfer and record-keeping	- Transfer procedures from the AMC to the HRHH
	- Patient record
	- Handover procedures during shifts

Monitoring and feedback cycles	- Steering group meetings
	- Weekly multidisciplinary meetings
	- Supervision by an AMC geriatric nursing specialist
	- Patient satisfaction questionnaire upon discharge from the transfer unit
	- Training and education
	- Management information system

On the transfer unit, the wheel is reinvented every day. Because people don't work according to the agreements, the implementation has been flawed. (respondent 6)

In the implementation literature, good preparation – involving the relevant people, developing a proposal for change and selecting a set of multifaceted strategies – is emphasised [23,24]. Even so, the necessity of an open culture for change and involved management is underscored. Contrasting these insights with our data revealed shortcomings in the implementation. First, the initial intermediate care model lacked a detailed implementation strategy. Various respondents who had worked in the unit from the start confirmed this. Second, it can be questioned whether all relevant people where involved soon enough. Chief executives confessed they involved the nursing home physician too late. Also, nursing assistants said they were insufficiently prepared. Third, there seemed to be a lack of communication, resulting in a 'closed organisational culture' and resistance to change. Finally, involvement by management was considered insufficient. There was criticism that supervision and control was too lax to bring about the desired change. This lack of management was partly due to discontinuities in leadership. Although four persons headed up the unit from the start, reorganisation of the entire residential home distracted chief executives. All these shortcomings were reflected in the following quotes:

At the start, there was very little idea of what might happen. (respondent 6)

They told us almost nothing about what was going to happen. I had the idea they didn't really know, either. (respondent 18)

A nursing home physician was represented in the project group, but it wasn't the physician who was going to do the job. I didn't think this was very smart.(respondent 2)

Staff members act differently. Different perceptions, different realities can be observed, but they're not tried out on each other or debated. (respondent 11)

Apart from the implementation, respondents were also concerned with the nursing staff mix. In their view, the staff was insufficiently qualified:

At this moment, I do not have a positive image of their expertise. (respondent 8)

Managers and staff at the HRHH attributed the lack of expertise to the shortage of skilled nurses. Local figures showed that in 2000, nursing homes and residential homes in Amsterdam had 56 vacancies per 1,000 nursing staff. This rate is the highest in the Netherlands [30]. The managers of the HRHH said they had enormous problems filling the vacancies. Respondents working in the AMC underscored this, but also questioned the chosen staff mix model of the transfer unit:

I was and still am disappointed in the nursing staff mix. The number of staff is okay, but on a ward where discharged hospital patients are admitted, just one RN supplemented by nursing assistants isn't enough. (respondent 2)

### Limitations

The process evaluation has its limitations. The large number of non-consenting patients (119 out of 189) has biased the quantitative results. Most of the non-consenting patients were unable or less inclined to participate in the study. We attribute this in part to a flawed informed consent procedure. Consenting patients had to fill out questionnaires immediately, which made various candidates less willing, especially the sicker ones. Consequently, healthier patients are over-represented in the sample. This explains why we could not verify the perception by respondents that the patient population was more seriously ill.

The validity of the qualitative findings is rather high, although some weaknesses were revealed. Because of our managerial evaluation perspective [18], three respondents seemed to give socially desirable answers. Furthermore, we had the impression that the evaluation became a management intervention in itself (*Hawthorne effect*). During the study we noticed that our framework of analysis, which was implicitly communicated during the interviews, was translated into management actions. Another weakness is that just one researcher conducted the interviews and analysis. Still, we were able to overcome these sources of bias because of the combination of methods, the purposive sampling procedure undertaken and the comprehensive triangulation achieved. The interviews therefore provide a credible exploration of the functioning of an intermediate care model in its local context.

Overall, the transportability of the findings to other settings is limited. As we conducted a process evaluation, findings only provide a detailed description of the intermediate care model in Amsterdam. Nevertheless, we assume that the accumulated impact of the model is not unique to the Amsterdam model. From this perspective, the findings provide a good starting point for developing and evaluating models elsewhere.

## Discussion

In the current climate of health care policy, many stakeholders advocate the development and implementation of intermediate care models. However, the widespread popularity of the concept is insufficiently supported by evidence. Intermediate care is still in need of evaluation, as the benefits and the deficits of the various models are ambiguous [1-4].

Nevertheless, available knowledge provides enough do's and don'ts for initiators who want to set up and implement intermediate care. Basically, initiators should be cautious. As our study shows, the accumulated impact of setting up such models may result in a 'bad practice'. To prevent this, initiators should adequately plan, organise and monitor intermediate care services. The following issues are important.

First, the relevance of an intermediate care model must be clear at the outset and supported by sound information. This was not the case in Amsterdam. The low intensity early discharge model was set up alongside two more intensive intermediate care models for stroke and orthopaedic patients. Consequently, the transfer unit was targeted towards a remnant and heterogeneous patient population within which the number of eligible candidates was smaller than expected. The straightforward conclusion is that one should know the size and profile of the target population. Apart from that, patients may view intermediate care as unacceptable. Little is known about this topic [31], and this is reflected in the call for more user involvement in intermediate care development [32]. Even so, this study indicates that the dynamic health status of candidates requires fast and flexible transition processes. Because the health status of patients can change rapidly, intermediate care services are appropriate for short periods during patient journeys. Moreover, poorly organised transition processes may increase the average LoS in the acute care setting which undermines achieving the goal of early discharging patients. So, detailed knowledge of the care needs, desires and size of the target population is necessary to justify the relevance of an intermediate care model.

Second, policy-makers and managers should pay attention to the dynamics resulting from organisational and financial incentives. These may influence a broadening of admission criteria to maximise bed occupancy rates, as shown in Amsterdam. In the literature, this phenomenon is also known as 'Roemer's law': a bed built is a bed filled [33-35]. Perverse incentives induce improper use of intermediate care beds, which results in another patient profile than initially anticipated. The changed patient profiles reported in various studies support this hypothesis [13,36,37]. Continuous monitoring of the patient profile in relation to bed occupancy rates is necessary to detect and overcome this phenomenon.

Third, intermediate care requires a minimum of nursing staff to guarantee quality of care delivery. Their skills should meet the needs of envisioned patients. This was inadequate in Amsterdam. Nursing assistants gave direct care to patients, while medical and registered nursing staff headed up the unit. This high percentage of direct care given by relatively unqualified staff is also reported in other studies. It is considered one of the main reasons for the absence of improved outcomes of intermediate care [13,36]. Other studies as well indicate that nursing staff *does *matter: those hospitals and nursing homes with the most highly qualified staff provide better care [38-40]. This underscores the importance of thoroughly considering nursing staff mix in intermediate care. However, it is difficult to determine what nursing staff mix is appropriate. There is no minimum standard available.

Fourth, the implementation of intermediate care needs to be given attention. It requires know-how and expertise. In Amsterdam, the initiators overestimated the ability of the staff of the residential home to develop and operate a transfer unit for early discharged patients. These homes provide minimal care services for elderly people in stable health. These homes are less prepared to deliver care to post-acute care patients. This is reflected in the less advanced stage of development of quality assurance activities in Dutch residential homes [41,42]. Initiators of collaborative intermediate care models should be aware of this pitfall and plan a comprehensive implementation strategy. Such a strategy must contain multiple approaches at different levels, tailored to specific settings and target groups [23,24]. Such a strategy should ensure that all requirements are met for delivering good care.

From a more general perspective, these issues promote a more rational and evidence-based management of intermediate care. It is acknowledged that the uptake of evidence in managerial practice could be better [43,44]. As intermediate care lacks a straightforward evidence base, managers run the risk of being persuaded by political willingness rather than by 'evidence'. Uninformed decision-making is dangerous and may ultimately harm patients; future research should fill the existing knowledge gaps. Steiner [1] identifies three key questions for intermediate care research: 1) Which services are best for which patients at which point? 2) Which professionals should be involved, doing what at which point? 3) What is the bottom line financially?

Our findings indicate that these key questions must be answered simultaneously. One cannot properly answer one of the key questions without knowing the answers to the other two. This implies that research designs should have a broad scope, which as our study illustrates, is at the expense of rigour. However, creative study designs are being promoted that try to balance rigour and validity by combining quantitative and qualitative approaches, purpose-collected and coincidental data, and multidisciplinary research perspectives [45,46]. These evaluative approaches provide a good foundation for developing the evidence base for intermediate care.

## Conclusion

We conclude that setting up a low intensive early discharge model of intermediate care between a university hospital and a residential home is less straightforward than was originally perceived by management, and that quality of care needs careful monitoring to ensure the change is for the better.

The AMC and HRHH management have taken on these lessons, and the findings of the process evaluation have been translated into management interventions: consistent use of discharge and admission criteria, increasing patient flows by working with a second general hospital in Amsterdam, increasing nursing and medical expertise on the ward, implementing specific nursing protocols and more systematic monitoring of care.

A combined quantitative and qualitative evaluation approach executed in close collaboration with the actors involved was helpful in revealing the underlying mechanism leading to the shortcomings.

## Competing interests

The author(s) declare that they have no competing interests.

## Authors' contributions

TP designed the qualitative study, conducted the semi-structured interviews, analysed the qualitative data, analysed all data, and primarily wrote the paper.

DMJD designed the whole study, drafted the main protocol, assisted with the quantitative data analysis, reviewed earlier drafts of the paper, and supervised the daily research activities

TFK collected quantitative data, was responsible for the quantitative data entry, did the quantitative analysis, and critically reviewed earlier drafts of the paper

TJ collected quantitative data, critically reviewed earlier drafts of the paper

NSK was responsible for completing the study and the supervision, designed the whole study, and critically reviewed earlier drafts of the paper

## Pre-publication history

The pre-publication history for this paper can be accessed here:


